# Endobiotic Ciliates from the Rumen of the European Bison *Bison Bonasus* (Linnaeus, 1758) from the Vologda Oblast of Russia

**DOI:** 10.1134/S001249662370045X

**Published:** 2023-10-13

**Authors:** O. A. Kornilova, L. V. Chistyakova, I. V. Gusarov

**Affiliations:** 1 Herzen State Pedagogical University, St. Petersburg, Russia; 2 Zoological Institute, Russian Academy of Sciences, St. Petersburg, Russia; 3 Emelianov North-Western Research Institute of Dairy and Meadow Pasture Farming, Vologda Research Center of the Russian Academy of Sciences, Vologda, Russia

**Keywords:** endobiotic ciliates, *Bison bonasus*, Ophryoscolecidae, Isotrichidae, Vologda

## Abstract

The fauna of endobiotic ciliates of the rumen of the European bison *Bison bonasus* from the Vologda oblast of Russia was investigated. In the studied bisons, 12 species of trichostomatids (Trichostomatia, Litostomatea) were found, ten of which were from the family Ophryoscolecidae and two species were from the family Isotrichidae. A high similarity of ciliate faunas in the rumen of different bison individuals in the studied population was noted. A comparative analysis of data on the species diversity and number of endobiotic ciliates in the rumen of various representatives of the genus *Bison* from different habitats was carried out.

Endobiotic ciliate fauna inhabiting the digestive tract of vertebrate animals is specific for different systematic groups of hosts from the rank of order and above [1, 2]. The species diversity of ciliate endobionts of ruminants includes members of two families: Ophryoscolecidae (Trichostomatia, Entodiniomorphida) and Isotrichidae (Trichostomatia, Vestibuliferida). Ciliates inhabit predominantly the anterior digestive tract of these mammals, but some species can also be found in the posterior parts of the intestine. The species diversity of endobiotic ciliates of ruminants is determined by the characteristics of the biology and ecology of the host [3]. The most important factors are the food diet of the host, the level of gregariousness, the degree of isolation of individual populations, and the presence of interactions with other ruminant species [2, 4, 5].

In 1991, as a part of the project on the preservation of *Bison bonasus* (Linnaeus, 1758) in Russia, an experiment was launched to establish a free-ranging population of bison in Vologda oblast [6, 7]. A group of three purebred bison came from the Bison Breeding Center (Prioksko-Terrasny Nature Reserve, Moscow oblast) and was released into natural environment. The animals have successfully adapted and produce fertile offspring. To replenish and improve the gene pool of the emerging herd, new animals are regularly imported and released from the Bison Breeding Center and the Oka Reserve (Ryazan’ oblast). To date, the bison population of Vologda oblast counts 108 individuals; it is the most northern free-living bison population in the world.

It is known that endobiotic ciliates take an active part in the digestive processes in ruminants [3, 5]. In this context, it is certainly relevant to study the fauna of endobiotic ciliates of bison inhabiting Vologda oblast. This study is of particular interest because it provides a possibility to observe the consequences of the so-called bottleneck effect on the ciliate community, considering that the host animal population developed from an extremely limited number of individuals.

This work presents for the first time the results of a detailed study of the species diversity of endobiotic ciliates from the rumen of the European bison *Bison bonasus* inhabiting Vologda oblast (Russia). A comparative analysis of available data on the structure of endobiotic ciliate communities from the digestive tract of members of the genus *Bison* Hamilton Smith, 1827 from different habitats was carried out.

## MATERIALS AND METHODS

The study was carried out in ciliates obtained from the rumen of two European bison females during the winter season: sample no. 1 was collected in December, 2015, and sample no. 2, in February, 2018. Both samples were collected in vicinity of the settlement Bol’shaya (Ust’-Kubenskii district of Vologda oblast; 60.06912° N, 39.33602° E). Since the bison population in Vologda oblast was created for the purpose of preserving these animals in Russia as a free-ranging herd, samples of rumen content can be obtained only in the case of death of host individuals. Samples no. 1 and 2 were collected from female bisons that died in 2015 following a lethal spine injury and in 2018 due to a car accident, respectively.

Samples were fixed in 10% formalin solution in a 1 : 1 ratio. Light microscopy and microphotography were carried out using the microscopes MBI-11, Altami-Invert-3 with a camera adapter, and Leica DM2500 with differential interference contrast and a Leica DFC495 digital camera (8.0 MP).

For the analysis of cell morphology, macronuclei were revealed using 0.1% methyl green solution in 1% acetic acid and Lugol’s solution. Ciliate abundance per 1 mL of rumen content was determined using the calibrated drop technique [8]. Identification of ciliate species was carried out according to the manuals by Dogiel [9], Kofoid and MacLennan [10], Williams and Coleman [3], and Dehority [11].

## RESULTS AND DISCUSSION

The bison habitat in Vologda oblast represents a mosaic of southern taiga lands. Its characteristic features are an extensive network of water bodies and diverse vegetation of trees, shrubs, and floodplain meadows, which provides a rich forage base for the animals. During the period of positive temperatures, bison consume green mass of natural grass and twig fodder and need no additional feeding. Tree fodder accounts for about 30% of the average annual diet of bison. This figure varies from 2% in summer and autumn months to 70% in late winter and early spring. The diet of bison differs sharply in different seasons and different times of day. In summer, they feed predominantly on herbaceous plants. While bison consume a great variety of plants, the basis of their diet is made up of cereals, composites, rosaceae, legumes, umbellifers, and willows. Importantly, the animals consume a certain number of poisonous plants, such as buttercups, wolf’s-bane, and other. In the cold season, bison feed mainly in forest habitats, preferring deciduous and mixed forest types. The basis of nutrition in the winter period is wood and twig fodder. In addition, during this time the animals receive supplementary feed in the form of mixed fodder and hay. The absence of open water sources in winter does not affect the viability of bison, because they successfully use snow to quench their thirst. Bison also have constant access to salt sources (stone salt lick for ungulates is regularly laid out in the forest).

**Fig. 1.**
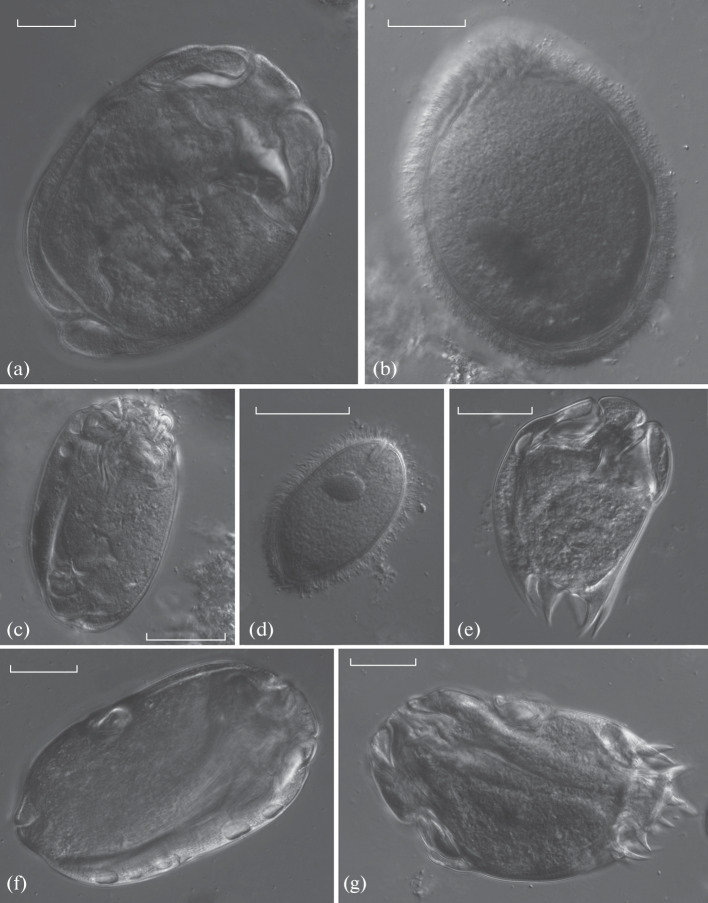
Endobiotic ciliates from the rumen of the European bison from Vologda oblast: (a) *Eudiplodinium maggii*, (b) *Isotricha prostoma*, (c) *Metadinium ypsilon*, (d) *Dasytricha ruminantium*, (e) *Diplodinium dentatum*, (f) *Elytroplastron bubali*, (g) *Ophryoscolex purkynjei*. Light microscopy, DIC*.* Scale bar, 30 µm.

**Fig. 2.**
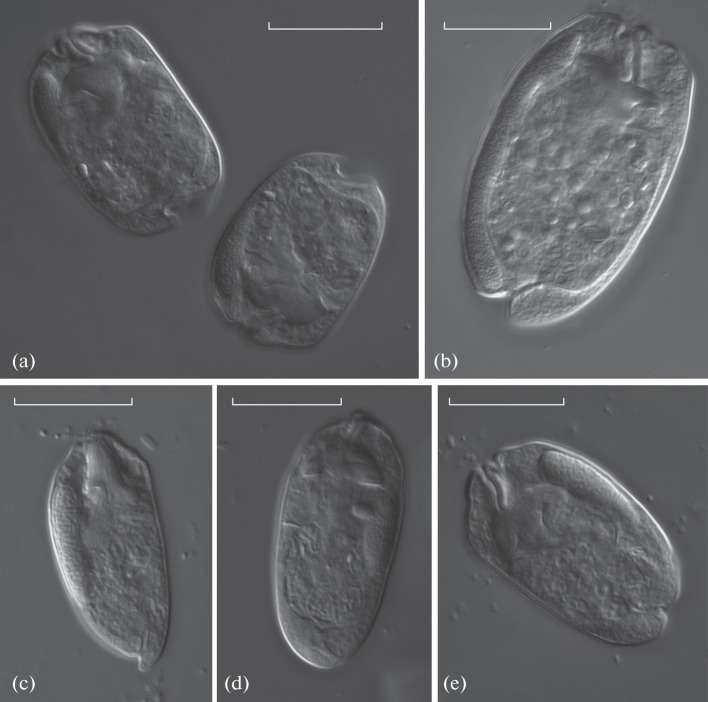
*Entodinium* ciliates from the rumen of the European bison from Vologda oblast: (a) *Entodinium* *lobosospinosum*, (b) *E. longinucleatum*, (c) *E. brevispinum*, (d) *E. elongatum*, (e) *E. simplex*. Light microscopy, DIC*.* Scale bar, 20 µm.

In the samples from the bison rumen, we identified 12 species of Ophryoscolecidae and Isotrichidae ciliates (Table 1; Figs. 1 and 2). The dominant group were members of the genus *Entodinium*: they constituted 68.4 and 69.2% of the total number of species in samples no. 1 and no. 2, respectively. Similar results (presented in Table 2 in comparison with our data) were also obtained by other authors [12–15]. According to the data from various studies, members of *Entodinium* accounted for 60–91.6% of the total number of species.

**Table 1.** Morphometric parameters of ciliates from the rumen of European bison from Vologda oblast (Russia)

No.	Species	Sample no. 1			Sample no. 2		
		Length	Width	Length to width ratio	Length	Width	Length to width ratio
1	*Entodinium* *longinucleatum* Dogiel, 1925	59.5 ± 0.62	36.1 ± 0.48	1.6–1.7	54.9 ± 0.71	38.4 ± 0.22	1.5–1.6
		54.6–63.3	34.5–40.3		51.8 – 63.3	34.5–43.1	
2	*E. lobosospinosum* Dogiel, 1925	46.0 ± 0.52	27.1 ± 0.44	1.6–1.8	46.4 ± 0.68	25.0 ± 0.24	1.6–1.8
		34.5–51.8	21.1–34.0		44.8–48.2	23.0–28.8	
3	*E. elongatum* Dogiel, 1927	44.4 ± 0.71	17.2 ± 0.43	2.5–2.7	48.0 ± 1.20	20.4 ± 0.64	2.3–2.7
		40.3–46.0	17.0–17.6		45.8–51.8	17.5–23.2	
4	*E. brevispinum* Kofoid & MacLennan, 1930	40.3 ± 0.54	17.4 ± 0.12	2.3–2.5	30.8 ± 0.64	18.8 ± 0.21	1.4–2.0
		34.5–43.1	17.2–17.5		28.6–34.8	17.1–20.1	
5	*E. simplex* Dogiel, 1927	36.8 ± 2.12	21.5 ± 1.44	1.7–1.8	38.4 ± 0.56	20.2 ± 1.50	1.7–2.0
		28.9–43.8	15.4–24.0		30.0–40.6	17.0–20.4	
6	*Diplodinium* *dentatum* (Stein, 1858)	86.3 ± 0.91	57.7 ± 0.82	1.3–1.7	79.9 ± 1.45	57.1 ± 0.93	1.3–1.5
		70.5–92.0	51.8–69.0		74.8–86.3	55.2–60.4	
7	*Eudiplodinium* *maggii* (Fiorentini, 1889)	177.3 ± 1.96	123.5 ± 1.60	1.4–1.7	164.4 ± 1.55	121.1 ± 2.16	1.2–1.5
		172.5–184.0	103.5–132.3		152.4–172.5	115.0–126.5	
8	*Metadinium* *ypsilon* (Dogiel, 1925)	92.0 ± 0.84	55.4 ± 1.64	1.5–1.6	96.0 ± 0.96	± 0.48	1.6–1.9
		80.5–109.3	48.9–69.2		91.8–103.8	50.8–63.3	
9	*Elytroplastron* *bubali* Dogiel, 1928	143.8 ± 2.94	42.0 ± 1.78	1.6–1.9	142.6 ± 1.90	86.4 ± 0.78	1.5–1.8
		120.8–161.0	63.3–92.0		138.0–149.5	80.5–97.8	
10	*Ophryoscolex* *purkynjei* (Fiorentini, 1889)	155.2 ± 1.81	91.3 ± 2.01	1.3–2.0	148.8 ± 2.43	80.4 ± 1.30	1.8–1.9
		143.8–178.3	69.0–115.0		138.0–172.5	71.9–92.2	
11	*Dasytricha* *ruminantium* Schuberg, 1888	57.5 ± 0.68	38.8 ± 0.72	1.3–2.2	61.6 ± 1.49	38.5 ± 0.58	1.3–1.9
		46.0–74.8	31.6–51.8		45.5–86.3	31.5–43.1	
12	*Isotricha prostoma* Stein, 1859	143.8 ± 3.12	95.6 ± 2.05	1.4–1.6	131.1 ± 2.75	90.4 ± 1.41	1.3–1.6
		115.0–161.0	74.8–106.4		120.0–139.2	80.2–97.9	

For both length and width, the data on the mean values (top line) and the min–max range (bottom line) are provided.

**Table 2.** Relative abundances and total number of ciliates of different genera in samples from the rumen of bison, according to different authors

Parameter	Lalueva et al., 2005	Gusarov et al., 2013	Kulakova et al., 2017	Kisidayova et al., 2021	Sample no. 1	Sample no. 2
Relative abundance of ciliate genera, %						
Ophryoscolecidae						
*Entodinium*	86.4–91.2	60.0–90.0	91.6	82.86	68.4	69.2
*Diplodinium*	2.6–7.6	3.5–18.0	3.4	0.86	5.3	7.5
*Eudiplodinium*	–	–	–	0.23	1.2	0.5
*Ostracodinium*	–	–	–	0.01	–	–
*Metadinium*	–	–	–	0.13	3.4	5.1
*Elytroplastron*	–	0.3–1.1	–	0.12	3.7	4.2
*Eremoplastron*	–	–	–	0.30	–	–
*Epidinium*	0.9	0.3–1.1	0.2	0.29	–	–
*Ophryoscolex*	0.9–1.1	0.7–2.2	0.3	0.03	1.7	1.4
Isotrichidae						
*Isotricha*	2.4–2.6	2.8–13.4	0.8	0.87	1.7	2.3
*Dasytricha*	1.1–8.0	3.1–14.6	3.7	14.36	14.6	9.8
				100	100	100
Number of ciliates per 1 mL of rumen content						
	85 000–142 500	205 000–891 250	94 792	105 279–463 181	254 400	266 200

Noteworthy is the fact that the communities of endobiotic ciliates in both female bison studied were nearly identical. This may be due to a potential close relationship of these specific animals (e.g., a mother and a daughter), as well as to general uniformity of the endobiotic fauna in this population of bison.

According to our data, the abundance of ciliates in bison rumen content was 254 400 and 266 200 individuals per 1 mL in samples nos. 1 and 2, respectively (Table 2). On the whole, these numbers are consistent with the results reported by other authors for European bison from various habitats (Table 2). According to the previously published data, the total abundance of ciliates in the rumen of bison from Vologda oblast ranged from 48 750 to 891 250 individuals per mL [12–14]. These authors noted that the abundance of ciliates in the rumen of host animals feeding mainly on woody fodder was significantly lower than in the rumen of bison whose diet consisted predominantly of twigs and grass. A relationship between ciliate abundance and host’s diet was demonstrated for many species of ruminants, including members of the genus *Bison* [15–17]. Since samples no. 1 and 2 were collected in the winter period, when bison feed mainly on tree branches, we suppose that the abundance of ciliates in these samples corresponds to the seasonal norm.

Ten of the twelve ciliate species identified in our work were also found in bison from the Białowieża Forest (Poland) [15]. A likely explanation of this fact is that the Vologda population of bison originates from the Polish population (it was from Białowieża Forest that the first purebred bison were imported in Russia in 1948–1951). However, the number of ciliate species found in bison from Poland (32) was significantly larger; as a consequence, the Czekanowski–Sørensen similarity index for the endobiont faunas of the bison populations from Białowieża Forest and Vologda oblast was only 0.45.

At the same time, two species of ciliates found in bison form Vologda oblast (*Entodinium elongatum* and *Metadinium ypsilon*) were absent in bison from Białowieża Forest. It is possible that these ciliates were acquired by bison generations after their arrival to Russia, or they might have been lost in the Polish population. The appearance of new ciliate species in the community may also be related to the importation of bison into the population with the purpose of enriching the gene pool. The herd was replenished with a total of 23 individuals imported from the Prioksko-Terrasny and the Okskii Nature Reserves in 2010 and 2017.

The fauna of endobiotic ciliates from the rumen of bison from Vologda oblast was described in previous studies [12–14]. These observations included seven genera of endobiotic ciliates: *Entodinium*, *Diplodinium*, *Epidinium*, *Ophryoscolex*, *Polyplastron*, *Isotricha*, and *Dasytricha* (species-level identification was not carried out). The ciliate communities were dominated by species of the genus *Entodinium*, a considerable part of the ciliates belonged to family Isotrichidae, and the other species were represented by some sporadic cells.

In the present study, we did not observe *Polyplastron* sp. but identified *Elytroplastron bubali*, which is listed under the genus name *Polyplastron* in the Dogiel’s manual [9]. In our opinion, it was also *Elytroplastron bubali* that was detected in previous works; it is a highly typical member of endobiotic fauna in bison. We did not detect any members of *Epidinium*; at the same time, we observed *Metadinium ypsilon*, which had not been described in bison previously. The endobiotic rumen community of the bison from Poland included a different species: *Metadinium esalqum* (Dehority, 1979) [15]. According to the study by Cedrola et al. [18] dedicated to the taxonomy of the genus *Metadinium*, the shape of the cell is basically the only trait that allows discriminating between these two species: it is rectangular in *M. esalqum* and oval in *M. ypsilon.* Considering that the level of polymorphism in Ophryoscolecidae is rather high, it seems plausible that these members of the genus *Metadinium* represent different morphotypes of the same species. This question requires further investigation.

In comparison to the endobiotic ciliate fauna of bison analyzed in the present work, the fauna of the European bison population from Poland, as well of American bison, exhibited significantly greater species diversity [15–17, 19]. Altogether, 32 species of ciliates were identified in European bison from Poland and at least 25 species were found in American bison (not all of the detected ciliates were identified on the species level). The limited species diversity of endobiotic ciliates observed in the bison from Vologda oblast may be related to the so-called bottleneck effect. The modern Vologda population of bison has developed from very few individuals, and the endobiotic species richness may be determined by the composition of the ciliate community of the initial herd. In addition, the species composition of ciliate communities can be affected by specific features of the bison forage base in the area, as well as by eventual presence of predator ciliate species.

Eadie [4] proposed that endobiotic ciliate communities of ruminants should be differentiated depending on the spectrum of dominant species. This approach was applied by Towne et al. [16, 17], who investigated the endobiotic ciliate fauna of the rumen of American bison *Bison bison* Linnaeus, 1858, as well as by Kišidayová et al. in their study of endobiotic ciliates of European bison [15]. According to Kišidayová et al. [15], in a majority of cases (56% of animals), the rumen of bison from Białowieża Forest contained ciliates of type B with typical members of the genera *Epidinium* and *Eudiplodinium.* In the other bison, the range of ciliate species was of a mixed type A + B with the genus *Ophryoscolex*, which differed from ciliate group A. Endobiotic ciliate communities from most studied members of the genus *Bison* from various habitats represented type B [15–17]. Communities of type A + B, as well as of type A, were observed in those cases where the host population was in contact with domestic cattle [16, 17]. The ciliate communities of bison from Vologda oblast can be classified as type A + B, since they include both a species typical for group A (*Ophryoscolex purkynjei*) and a species typical for group B (*Eudiplodinium* *maggii*).
