# Erratum to: **Endobiotic Ciliates from the Rumen of the European Bison** ***Bison Bonasus*** **(Linnaeus, 1758) from the Vologda Oblast of Russia**

**DOI:** 10.1134/S0012496623050034

**Published:** 2024-01-25

**Authors:** O. A. Kornilova, L. V. Chistyakova, I. V. Gusarov

**Affiliations:** 1 Herzen State Pedagogical University, St. Petersburg, Russia; 2 Zoological Institute, Russian Academy of Sciences, St. Petersburg, Russia; 3 Emelianov North-Western Research Institute of Dairy and Meadow Pasture Farming, Vologda Research Center of the Russian Academy of Sciences, Vologda, Russia

The article “Endobiotic Ciliates from the Rumen of the European Bison *Bison Bonasus* (Linnaeus, 1758) from the Vologda Oblast of Russia,” written by O.A. Kornilova, L.V. Chistyakova, and I.V. Gusarov, was originally published electronically in Springer-Link on October 13, 2023 without Open Access. After publication in volume 511, pages 206–212, the authors decided to make the article an Open Access publication. Therefore, the copyright of the article has been changed to © The Author(s) 2023 and the article is forthwith distributed under the terms of a Creative Commons Attribution 4.0 International License (http://creativecommons.org/licenses/by/4.0/, CC BY), which permits use, duplication, adaptation, distribution and reproduction of a work in any medium or format, as long as you cite the original author(s) and publication source, provide a link to the Creative Commons license, and indicate if changes were made.

